# From hypotonic maintenance fluid to severe hyponatremia: a case report

**DOI:** 10.1186/s13256-021-02889-0

**Published:** 2021-06-07

**Authors:** M. Denis, A. Di Giacomo, E. Lacotte, F. Porcheret, N. Letouzé, B. Lauzier, I. Goyer, D. Brossier

**Affiliations:** 1 Pediatric Intensive Care Unit, CHU de Caen, 14000 Caen, France; 2 Pediatric Intensive Care Unit, CHU de Nantes, 44000 Nantes, France; 3 CNRS, INSERM, l’institut du thorax, Université de Nantes, CHU Nantes, 44000 Nantes, France; 4 Pediatric Department, CHU de Rouen, 76000 Rouen, France; 5 Medical School, Université de Rouen, 76000 Rouen, France; 6 Pediatric Nephrology, CHU de Nantes, 44000 Nantes, France; 7 Institut du thorax, INSERM, CNRS, UNIV Nantes, 44000 Nantes, France; 8 Department of Pharmacy, CHU de Caen, 14000 Caen, France; 9 Medical School, Université Caen Normandie, 14000 Caen, France; 10 Service de reanimation pédiatrique, CHU de Nantes, 4ème étage bâtiment HME, 38 boulevard Jean-Monnet, 44093 Nantes Cedex 1, France

**Keywords:** Hyponatremia, Hypotonic fluid, Intensive care, Isotonic fluid

## Abstract

**Background:**

The principles for maintenance intravenous fluid prescription in children were developed in the 1950s. These guidelines based on the use of hypotonic solutions have been challenged regularly for they seem to be associated with an increased risk of hospital-acquired hyponatremia.

**Case presentation:**

We report the case of a 4-week-old Caucasian child admitted for acute bronchiolitis who received hypotonic maintenance fluids and developed severe hyponatremia (94 mmol/L) with hyponatremic encephalopathy.

**Conclusion:**

This clinical situation can serve as a reminder of the latest recommendations from the American Academy of Pediatrics regarding the use of intravenous fluids that promote the use of isotonic fluids in children.

## Background

Maintenance intravenous fluids (MIVF) prescription practices are highly variable among pediatricians [1]. For decades, MIVF prescriptions were based on the 1957 Holliday and Segar recommendations [2]. These guidelines recommended the use of hypotonic fluids as a standard of practice for MIVF in children. Since 1957, this dogma has been constantly challenged for it appears to be associated with severe episodes of hospital-acquired hyponatremia [3, 4]. Besides hyponatremia, wrongful usage of intravenous fluid therapy can be associated with pericardial effusion and ascites, which can also be caused or enhanced by malignancy and sepsis [5–7]. Severe hyponatremia is defined as a plasma sodium level < 125 mmol/L and is associated with hyponatremic encephalopathy [8, 9], a spectrum of symptoms related to cerebral edema. Severe hyponatremia is a medical emergency that can lead to irreversible brain injury or death, if not managed promptly [8]. To help avoid this critical iatrogenic complication, the American Academy of Pediatrics published a guideline regarding MIVF in 2018, making only one and very simple recommendation: prescribe isotonic intravenous fluids in children [10]. Despite its straightforwardness, this simple recommendation remains neglected. We present the case of a 4-week-old child admitted for acute bronchiolitis who received hypotonic MIVF and then developed hyponatremic encephalopathy.

## Case presentation

**Fig. 1:**
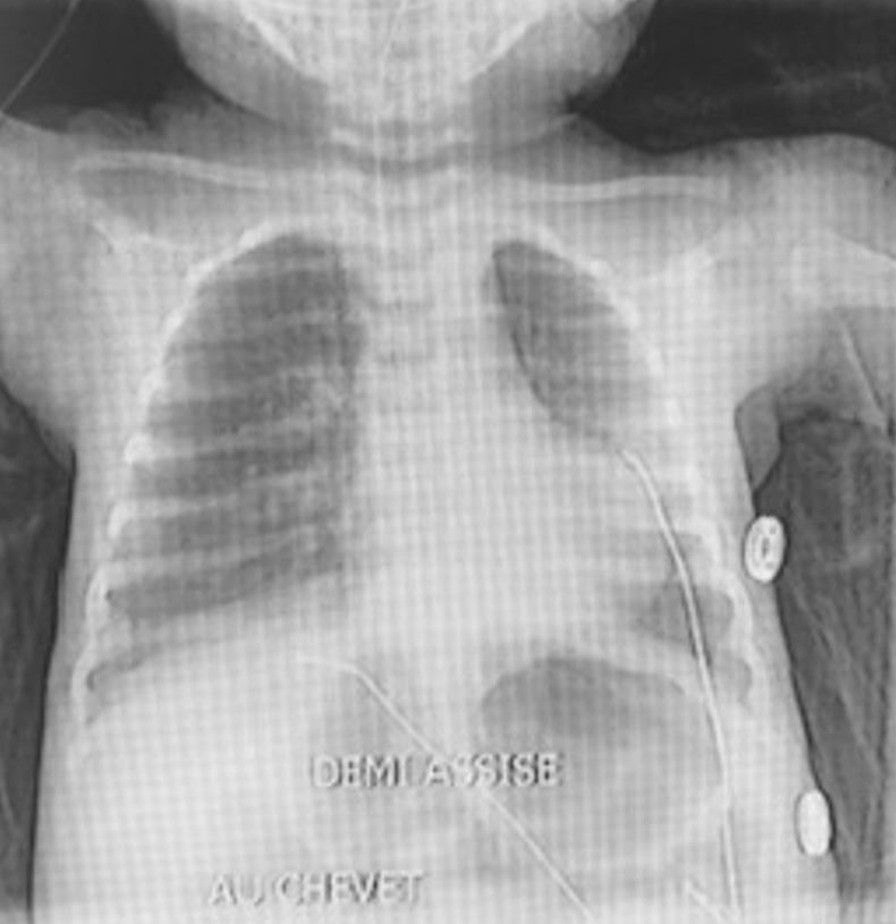
Chest X-ray upon admission to the pediatric ward

**Fig. 2:**
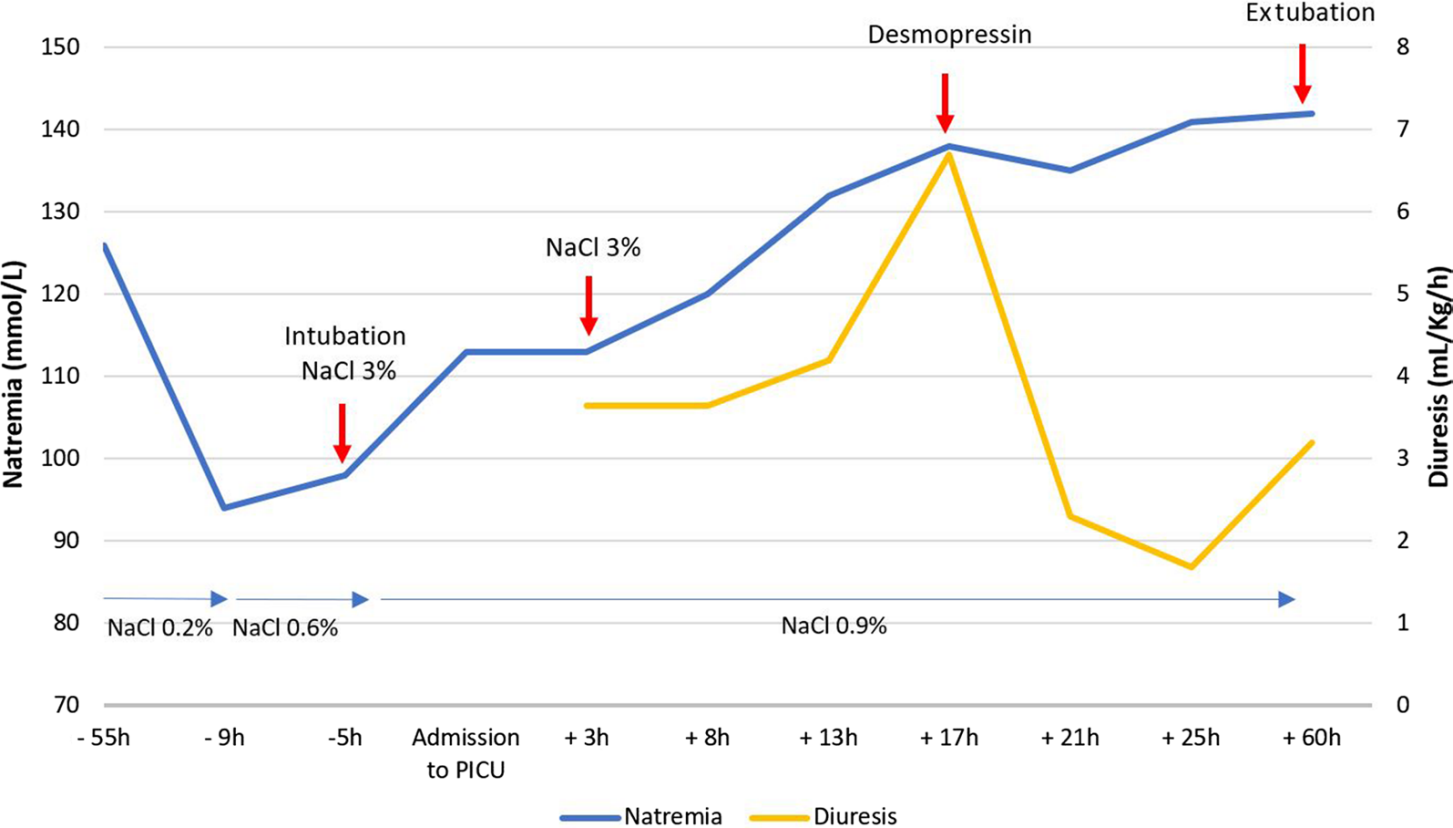
Natremia and diuresis over time before and after PICU admission. h: hours; NaCl 3%: hypertonic solution NaCl 3% (3 mL/Kg); desmopressin (0.1 mg/kg); NaCl 0.2%: maintenance intravenous fluid containing 2 g/L of NaCl; NaCl 0.6%: maintenance intravenous fluid containing 6 g/L of NaCl; NaCl 0.9%: maintenance intravenous fluid containing 9 g/L of NaCl

A 4-week-old, 2.8 kg Caucasian girl presented to the emergency department of a general hospital with a 1-day history of upper airway infection and poor feeding. She was born at 35 weeks of gestational age with a birth weight of 2.380 kg, Apgar 10, in a context of emergency C-section subsequent to preeclampsia and had no risk factor for mother-to-infant infection. She was admitted to the pediatric ward with a diagnosis of mild bronchiolitis with signs of rhinitis and reduced food intake, but showed no sign of dehydration. Upon admission, the patient was apyretic (37.5 °C), heart rate (HR) was at 172 beats per minute, blood pressure (BP) was 90/50 mmHg, and respiratory rate (RR) was 32 breaths/minute. She had no signs of hypoperfusion, and heart sounds were regular, with no audible murmur. Respiratory examination showed bilateral crackles, with moderate respiratory distress signs, and saturation was 100% under oxygen therapy 0.5 L/minute. She had moderate axial hypotonia, normal cries, and normotensive anterior fontanelle. There were no other findings on physical examination. Chest X-ray showed a chest distension without apparent infectious site (Fig. 1). Respiratory panel test was negative, including for respiratory syncytial virus (RSV) and influenza, as well as aerobic and anaerobic blood cultures. Blood tests at admission reported hyponatremia and hyperkalemia (Na^+^ 126 mmol/L, K^+^ 5.6 mmol/L, bicarbonate 22.3 mmol/L, creatinine 20 μmol/L, urea 5.4 mmol/L, white blood cells (WBC) 9.2 × 10^9^/L, hemoglobin (Hb) 10.9 g/dL, platelets (Plt) 553 × 10^9^/L, C-reactive protein (CRP) < 5 mg/L) (Fig. 2). She received continuous intravenous infusion of hypotonic fluid (sodium chloride 34 mmol/L, potassium 20 mmol/L, calcium 2.26 mmol/L, and glucose 5%) at a rate of 122 mL/kg/day, plus enteral feeding via nasogastric tube (130 mL/kg/day), supplemental oxygen 0.5 L/minute, respiratory physiotherapy, and oral betamethasone (0.375 mg once daily for 2 days).

**Fig. 3:**
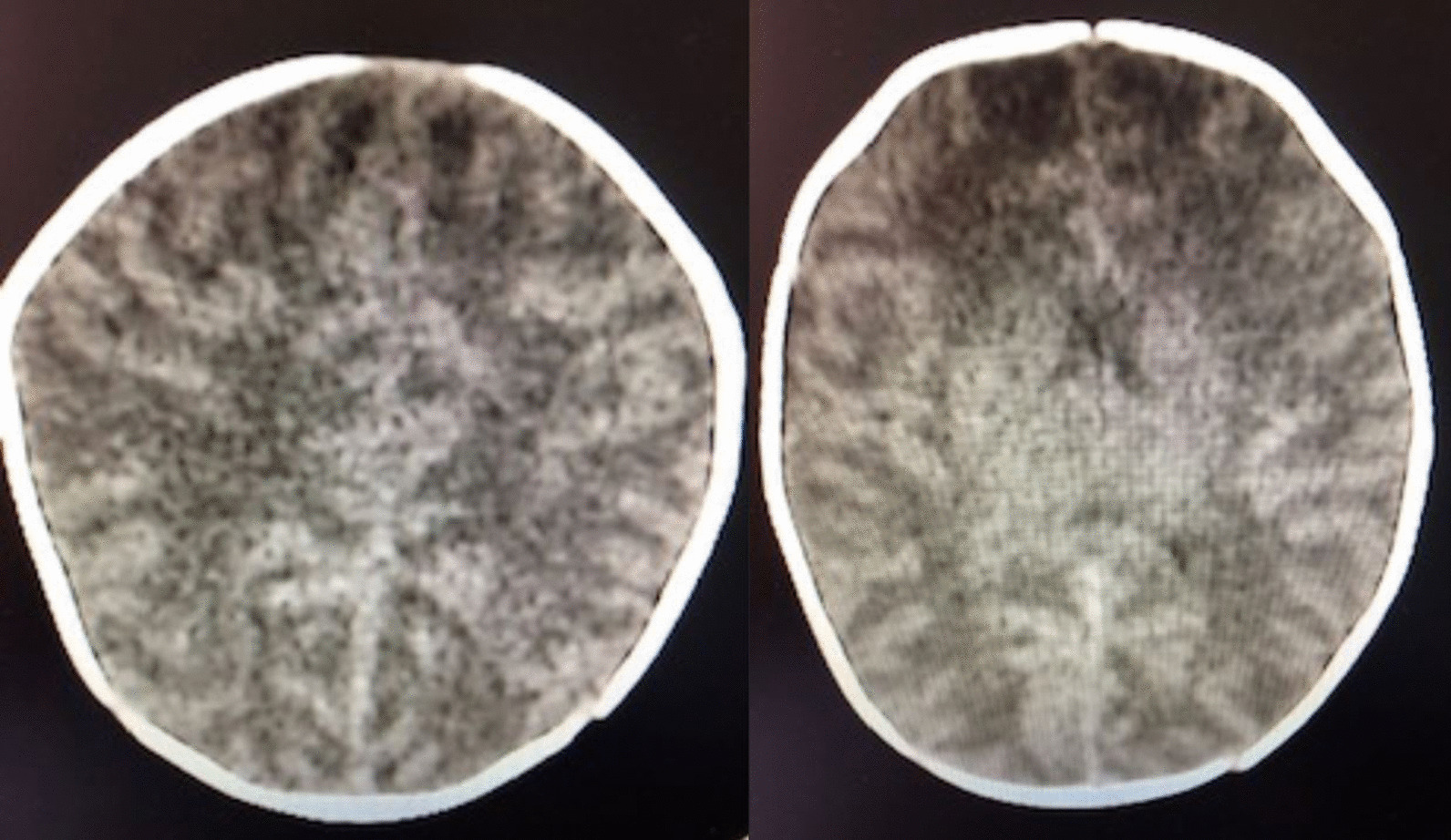
Cerebral tomodensitometry depicting widespread cerebral edema

After 48 hours, she was found drowsy and hypotonic, still reactive to stimulation with no other sign of intracranial hypertension. She presented respiratory distress, intermittent desaturation, hypothermia (35.5 °C), and normal hemodynamic parameters (HR 140 beats per minute and BP 95/50 mmHg). The blood tests showed: glycemia 6.10 mmol/L, Na^+^ 94 mmol/L, K^+^ 5.2 mmol/L, Cl^−^ 67 mmol/L, osmolality 194.1 mmol/kg, creatinine 10 μmol/L, urea 3 mmol/L, WBC 18.4 × 10^9^/L, Hb 10.8 g/dL, Plt 734 × 10^9^/L, CRP < 5 mg/L and urine tests: Na^+^ 48 mmol/L, K^+^ 50 mmol/L, Cl^−^ 102 mmol/L and osmolarity 456 mosm/L. NaCl was added to the MIVF (total Na^+^ content: 100 mmol/L), total intravenous intakes were restricted to 85 mL/kg/day, and feedings were stopped. Cerebral tomodensitometry showed widespread cerebral edema (Fig. 3). Four hours later, the blood tests showed persistent severe hyponatremia (98 mmol/L). Patient transfer to the Pediatric Intensive Care Unit (PICU) was then decided. Upon arrival of the pediatric transport team, the child was unconscious (Glasgow Coma Scale: 8) and presented respiratory pauses and hemodynamic symptoms of intracranial hypertension (HR 115 bpm, BP 110/60 mmHg). Auscultation revealed a decrease of the left vesicular murmur, and hemodynamics remained stable. No other abnormal clinical signs were found. She immediately received osmotherapy (3 mL/kg of NaCl 3% w/v). She was then sedated with propofol (3 mg/kg) and paralyzed with succinylcholine (1 mg/kg) for rapid sequence intubation and intubated with a 3.5-cuffed tracheal tube. Afterwards, she received isotonic MIVF (dextrose 5% with NaCl 145 mmol/L) with a total intake restricted to 80 mL/kg/day. Upon PICU admission, she was sedated with continuous infusions of midazolam (30 μg/kg/hour) and morphine (30 μg/kg/hour), without any clinical sign of intracranial hypertension.

Three hours after admission, transcranial Doppler ultrasound (TDU) showed elements of intracranial hypertension (pulsatility index (PI): 1.40, diastolic velocity (*V*_d_): 16 cm/second). TDU was normalized (PI: 1.02, *V*_d_: 30 cm/second) after a second IV bolus of NaCl 3% w/v (3 mL/kg).

Thirteen hours postadmission, the patient presented with transient polyuria (5.4 mL/kg/hour) associated with a decrease in urinary density to 1.005 g/mL, urinary Na^+^ < 10 mmol/L, and a rapid increase in natremia (Fig. 2). Intravenous desmopressin 0.1 μg/kg was given to control polyuria and stabilize natremia. Subsequently, diuresis and natremia normalized with isotonic MIVF and fluid restriction for 2 days until resumption of enteral feedings.

Amplitude-integrated electroencephalography (aEEG) did not report any comitial activity and sedation was weaned 24 hours after admission. The infant showed signs of awakening. She was extubated 2 days after admission, and then received supplemental oxygen for 5 days and systemic steroids (intravenous dexamethasone 0.15 mg/kg every 6 hours for 24 hours) for postextubation laryngeal stridor.

Three days after admission, the child’s EEG reported elements of cerebral suffering and some left central acute elements for which epileptic origin was not confirmed. EEG performed on day 6 was normal. Magnetic resonance imaging (MRI), performed on day 4, showed significant regression of cerebral vasogenic edema with multiple small areas of supratentorial cytotoxic edema, whose distribution did not match any typical aspect of osmotic demyelination syndrome (ODS) and were imputed to ischemic injuries. This lack of ODS-related lesion was later confirmed by another MRI 6 months later.

SIAD assessment was performed with elimination of dysthyroidism [TSH 3.16 mUI/L (N)] and adrenal insufficiency [8 a.m. cortisol: 52.3 µg/L (N)], but antidiuretic hormone dosage was not available. Blood samples remained normal afterwards.

**Fig. 4:**
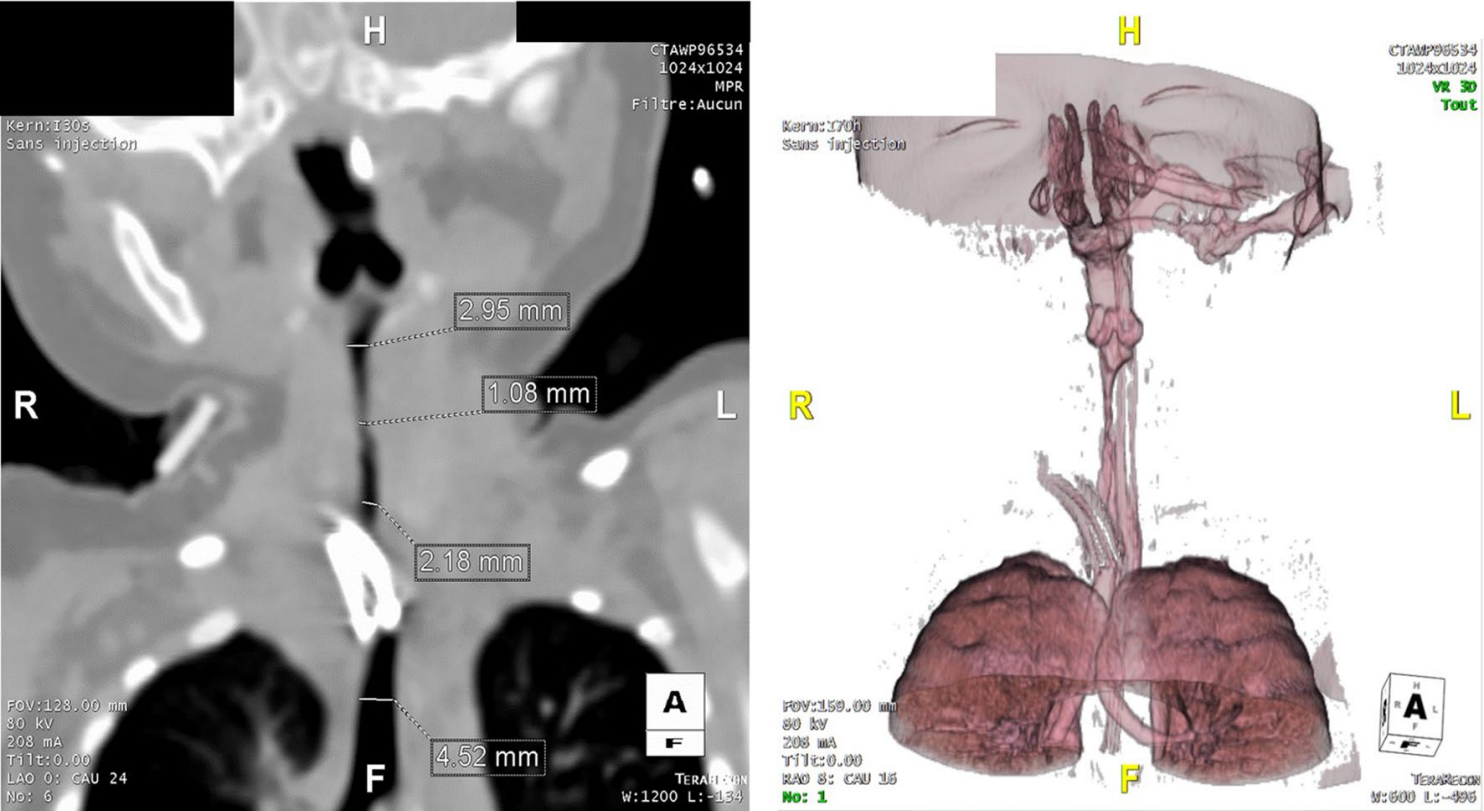
Tomodensitometry and three-dimensional (3D) reconstruction of the subglottic stenosis

Eight days after PICU admission, neurological examination was normal. The infant was transferred to the general pediatric ward on day 8 and discharged home on day 12. In the weeks following discharge, she presented with feeding difficulties and suffered episodes of acute discomfort with desaturation, bradycardia, and laryngeal spasm requiring rehospitalization in the pediatric ward and then in the PICU. Upon admission, HR was at 175 bpm, BP 120/68 mmHg, RR 40/minute, and saturation 100% under high-flow oxygen therapy with FiO_2_ 30%. She had inspiratory dyspnea with severe respiratory distress, and other clinical signs were normal. Faced with a serious acute airway obstruction episode and an inability to intubate, urgent tracheostomy was performed. A subglottic stenosis was later found (Fig. 4). A tracheostomy remained in place until the age of 14 months. She had multiple dilations of the subglottic stenosis with good results. At 2 years of age, she has a normal neurological clinical examination. She suffers from moderate asthma and is fed by a gastrostomy because of an orality disorder.

## Discussion

Isotonic maintenance intravenous fluid is recommended for hydration of hospitalized children [10]. We report here the case of a 4-week-old child who received hypotonic intravenous fluid and experienced secondary hyponatremia as well as dramatic neurological and respiratory complications, which led to a 1-year hospitalization period. This case demonstrates that following the guidelines regarding the use of isotonic fluid therapy is mandatory.

Over the past 60 years, infants and children received hypotonic MIVF as a standard of care, according to outdated recommendations that were based on theoretical calculations that were never validated in clinical trials. Even though numerous studies since the late 1990s have confirmed that hypotonic MIVF contributes to hospital-acquired hyponatremia [3, 4], these solutions are still commonly used in children owing to a fear of hypernatremia, hyperchloremic acidosis, or fluid overload [1, 11, 12]. The use of hypotonic intravenous solutions is all the more inappropriate since hospitalized children commonly present with SIAD [13] and reports of death or permanent neurological impairment arising from iatrogenic hyponatremia in both children and adults are documented [14]. The National Patient Safety Agency in the UK [15], the Institute for Safe Medication Practices of Canada [14], and the USA [16] warned about the dangers of infusing hypotonic intravenous fluids in children. This case outlines once again the importance of updating anchored hazardous clinical practices according to actual recommendations. The American Academy of Pediatrics published a clinical practice guideline in December 2018 strongly recommending that patients aged from 28 days to 18 years receive isotonic solutions when MIVF is needed [10] (Table 1). The strength of this recommendation should overcome the remaining concerns about the safety of isotonic MIVF in acutely ill children. Isotonic saline solutions have shown a protective effect against hyponatremia [17–19] without any significant association with deleterious effects such as fluid overload, hypernatremia, or phlebitis [20]. Besides, the amount of fluid intake should be adapted to every patients’ clinical situation, for there is no one-size-fits-all formula [11]. For example, one of the main causes of acquired hyponatremia during hospitalization in pediatrics is SIAD [11]. SIAD is characterized by excessive arginine-vasopressine (AVP) secretion leading to free-water retention that induces a decrease in plasmatic osmolarity associated with intracellular overhydration [9]. SIAD management essentially involves fluid intake restriction (600 mL/m^2^/day) [13] and sufficient sodium intake (6–10 g/day) to compensate for relative hypovolemia induced by intracellular fluid shift and associated reduction in plasma water volume [9]. Pediatricians should also realize that fluid overload is the consequence of the total amount of fluid intake more than the fluid’s tonicity, as is still commonly thought [11].

**Table 1:** Available maintenance intravenous fluid solution and necessary additives

Solution	Composition	Osmolarity (mOsmol/L)	Necessary additives for use in children (glucose 4–5% + near isotonic)	Estimated final composition	Estimated final osmolarity (mOsmol/L)
Human plasma	Glucose 3.5–6.1 mmol/L	308			
	Na^+^ 135–144 mmol/L				
	Cl^−^ 95–105 mmol/L				
	K^+^ 3.5–5.3 mmol/L				
	Bicarbonate 23–30 mmol/L				
	Ca^2+^ 2.2 mmol/L				
	Mg^2+^ 0.8–1.2 mmol/L				
Normal saline^a^	Na^+^ 154 mmol/L	308	80 mL of glucose 30% per 500 mL	Glucose 4.1%	499
	Cl^−^ 154 mmol/L			Na^+^ 133 mmol/L	
				Cl^−^ 133 mmol/L	
Normal saline G5%^a^	Glucose 5%	580	Nothing to add, but unbalanced in anions, and does not contain potassium or calcium		
	Na^+^ 154 mmol/L				
	Cl^−^ 154 mmol/L				
Ringer’s lactate^MD,a,b^	Na^+^ 130 mmol/L	253	80 mL of glucose 30% per 500 mL	Glucose 4.1%	452
	K^+^ 4 mmol/L			Na^+^ 112 mmol/L	
	Ca^2+^ 1.4 mmol/L			K^+^ 3.5 mmol/L	
	Cl^−^ 108 mmol/L			Ca^2+^ 1.2 mmol/L	
	Lactate 27 mmol/L			Cl^−^ 93 mmol/L	
				Lactate 23 mmol/L	
Ringer’s lactate G5%^MD,a,b^	Glucose 5%	525	Nothing to add, but slightly hypotonic		
	Na^+^ 130 mmol/L				
	K^+^ 4 mmol/L				
	Ca^2+^ 1.5 mmol/L				
	Cl^−^ 109 mmol/L				
	Lactate 28 mmol/L				
Hartmann’s solution^MD,a,b^	Na^+^ 131 mmol/L	278	80 mL of glucose 30% per 500 mL	Glucose 4.1%	466
	K^+^ 5.4 mmol/L			Na^+^ 113 mmol/L	
	Ca^2+^ 1.8 mmol/L			K^+^ 4.7 mmol/L	
	Cl^−^ 111.7 mmol/L			Ca^2+^ 1.6 mmol/L	
	Lactate 27.8 mmol/L			Cl^−^ 96.3 mmol/L	
				Lactate 24 mmol/L	
Ringer’s acetate^MD,a,b^	Na^+^ 137 mmol/L	291	80 mL of glucose 30% per 500 mL	Glucose 4.1%	475
	K^+^ 4 mmol/L			Na^+^ 118 mmol/L	
	Ca^2+^ 1.65 mmol/L			K^+^ 3.5 mmol/L	
	Mg^2+^ 1.25 mmol/L			Ca^2+^ 1.4 mmol/L	
	Cl^−^ 110 mmol/L			Mg^2+^ 1.1 mmol/L	
	Acetate 36.8 mmol/L			Cl^−^ 94.8 mmol/L	
				Acetate 31.7 mmol/L	
ELOMEL Isoton^MD,a,b^	Na^+^ 140 mmol/L	302	80 mL of glucose 30% per 500 mL	Glucose 4.1%	485
	K^+^ 5 mmol/L			Na^+^ 121 mmol/L	
	Mg^2+^ 1.5 mmol/L			K^+^ 4.3 mmol/L	
	Ca^2+^ 2.5 mmol/L			Mg^2+^ 1.3 mmol/L	
	Cl^−^ 108 mmol/L			Ca^2+^ 2.2 mmol/L	
	Acetate 45 mmol/L			Cl^−^ 93.1 mmol/L	
				Acetate 38.8 mmol/L	
Plasma-Lyte 148^MD,a,b^ Normosol^MD,a,b^ Isolyte^MD,a,b^	Na^+^ 140 mmol/L	294	80 mL of glucose 30% per 500 mL	Glucose 4.1%	479
	K^+^ 5 mmol/L			Na^+^ 121 mmol/L	
	Mg^2+^ 1.5 mmol/L			K^+^ 4.3 mmol/L	
	Cl^−^ 98 mmol/L			Mg^2+^ 1.3 mmol/L	
	Acetate 27 mmol/L			Cl^−^ 84.5 mmol/L	
	Gluconate 23 mmol/L			Acetate 23.3 mmol/L	
				Gluconate 19.8 mmol/L	
Plasmalyte 148 G5%^MD,a,b^	Glucose 5%	572	Nothing to add, but dose not contain calcium		
	Na^+^ 140 mmol/L				
	K^+^ 5 mmol/L				
	Mg^2+^ 1.5 mmol/L				
	Cl^−^ 98 mmol/L				
	Acetate 27 mmol/L				
	Gluconate 23 mmol/L				
Isofundine^MD,a,b^ Ringerfundine^MD,a,b^ Sterofundine^MD,a,b^	Na^+^ 145 mmol/L	309	80 mL of glucose 30% per 500 mL	Glucose 4.1%	499
	K^+^ 4 mmol/L			Na^+^ 125 mmol/L	
	Ca^2+^ 2.5 mmol/L			K^+^ 3.5 mmol/L	
	Mg^2+^ 1 mmol/L			Ca^2+^ 2.15 mmol/L	
	Cl^−^ 127 mmol/L			Mg^2+^ 0.9 mmol/L	
	Acetate 24 mmol/L			Cl^−^ 109 mmol/L	
	Malate 5 mmol/L			Acetate 20.7 mmol/L	
				Malate 4.3 mmol/L	
Isopedia^MD,a,b^	Glucose 1%	351	60 mL of glucose 30% per 500 mL	Glucose 4.2%	495
	Na^+^ 140 mmol/L			Na^+^ 125 mmol/L	
	K^+^ 4 mmol/L			K^+^ 3.6 mmol/L	
	Ca^2+^ 1 mmol/L			Ca^2+^ 0.9 mmol/L	
	Mg^2+^ 1 mmol/L			Mg^2+^ 0.9 mmol/L	
	Cl^−^ 118 mmol/L			Cl^−^ 107 mmol/L	
	Acetate 30 mmol/L			Acetate 27 mmol/L	
Glucidion G5^MD^	Glucose 5%	460	10 mL of NaCl^−^ 2 g/10 mL per 500 mL	Glucose 4.9%	585
OsmotanG5^MD^	Na^+^ 68.4 mmol/L			Na^+^ 136 mmol/L	
BionolyteG5^MD^	K^+^ 26.8 mmol/L			K^+^ 26.2 mmol/L	
Polyionique G5^MD^	Cl^−^ 95.2 mmol/L			Cl^−^ 163 mmol/L	
Dextrion G5%^MD^	Glucose 5%	393	15 mL of NaCl^−^ 2 g/10 mL per 500 mL	Glucose 4.8%	582
	Na^+^ 34.2 mmol/L			Na^+^ 136 mmol/L	
	K^+^ 20.1 mmol/L			K^+^ 19.5 mmol/L	
	Ca^2+^ 2.23 mmol/L			Ca^2+^ 2.2 mmol/L	
	Cl^−^ 54.3 mmol/L			Cl^−^ 156 mmol/L	

*Na*, sodium; *K*, potassium; *Cl*, chlorine; *Ca*, calcium; *Mg*, magnesium

^a^isotonic fluid

^b^balanced fluid

The patient’s natremia was rapidly corrected when hypertonic saline infusion was started, with an increase of 15 mmol/L in the first 5 hours (3 mmol/L/hour), and an overall increase of 40 mmol/L in the first 24 hours. Management of acute hyponatremia should be based on symptom severity rather than on sole natremia [13]. Recommended treatment for acute symptomatic hyponatremia ranges from 1 to 3 mL/kg (0.5 to 1.5 mmol/kg) of intravenous NaCl 3% w/v administered over 10–15 minutes [13, 21, 22]. Additional boluses are to be administered if the patient’s condition does not improve, with a target increase of 5 mmol/L in sodium concentration in the first 1–2 hours [13] to avoid ODS [21, 22]. ODS remains extremely rare and is more likely to occur in patients with concomitant risk factors as well as in the context of chronic hyponatremia [23]. The correction in the first 48 hours should not exceed 15–20 mmol/L [13]. In this case, the natremia correction rate was further accelerated by the temporary polyuria that appeared a few hours after initiation of the hypertonic infusion. Desmopressin administration allowed for a small reduction in the sodium correction rate. Despite the persistence of inappropriate AVP secretion, after a few days, there was an increase in diuretic activity with a decrease in urinary osmolarity [9]. This physiological response, which varies according to the individual and the etiology of hyponatremia, intends to maintain homeostasis and counteract the harmful effects of the water retention from SIAD [9].

## Conclusion

This case highlights the importance of using isotonic solutions in children whenever MIVFs are required. It is an occasion to remind pediatricians of the American Academy of Pediatrics’ recommendations regarding the use of isotonic MIVF in children.

## Data Availability

All data supporting the findings of this article are included in the manuscript.

## Abbreviations

aEEG: Amplitude-integrated electroencephalography
BP: Blood pressure
Hb: Hemoglobin
HR: Heart rate
MIVF: Maintenance intravenous fluids
MRI: Magnetic resonance imaging
ODS: Osmotic demyelination syndrome
PICU: Pediatric Intensive Care Unit
PI: Pulsatility Index
Plt: Platelets
RR: Respiratory rate
SIAD: Syndrome of inappropriate antidiuresis
TDU: Transcranial Doppler ultrasound
Vd: Diastolic velocity
WBC: White blood cells
